# Delayed diagnosis and treatment of Sheehan syndrome: a case report

**DOI:** 10.1016/j.xfre.2026.03.002

**Published:** 2026-03-13

**Authors:** Sara G. Vargo, Nicholas J. Thomas, Katherine S. Brito, Naghma A. Farooqi, David Chia

**Affiliations:** a School of Medicine, University of California, San Francisco, San Francisco, California; b Department of Medicine, University of California, San Francisco, San Francisco, California; c Department of Obstetrics, Gynecology, & Reproductive Sciences, University of California, San Francisco, San Francisco, California

**Keywords:** Sheehan syndrome, postpartum hypopituitarism, postpartum hemorrhage, hormone replacement therapy

## Abstract

**Objective:**

To report a case of Sheehan syndrome diagnosed and treated nearly 2 decades after a postpartum hemorrhage.

**Design:**

Case report.

**Subjects:**

A 45-year-old woman presenting with nonspecific symptoms found to have severe hyponatremia and panhypopituitarism 1 month after immigrating from China and 17 years after a postpartum hemorrhage.

**Exposure:**

Hormone replacement with hydrocortisone, levothyroxine, and combined oral contraceptive pills.

**Main Outcome Measures:**

Clinical and laboratory assessment of central adrenal insufficiency, hypothyroidism, and hypogonadotropic hypogonadism after initiation of treatment.

**Results:**

After initiation of hydrocortisone and levothyroxine, the hyponatremia and nonspecific symptoms, including fatigue, dyspnea, nausea, vomiting, myalgias, and brain fog, improved rapidly. Pituitary magnetic resonance imaging demonstrated markedly reduced pituitary volume. Dual-energy x-ray absorptiometry scanning revealed osteopenia. The patient has had 9 months of treatment with cyclic combined oral contraceptive pills without hormonal withdrawal bleeding. Overall, she reports significant improvement in quality of life.

**Conclusion:**

Postpartum hypopituitarism is widely underdiagnosed with nonspecific presentations and limited access to care contributing to diagnostic delay. Clinicians must maintain a high index of suspicion to diagnose hypopituitarism after a postpartum hemorrhage, even years after delivery. Early initiation of hormone replacement therapy is essential in patients with postpartum hypopituitarism for protection of cardiovascular and bone health.

## Introduction

Postpartum hypopituitarism, or Sheehan syndrome, is a rare complication of postpartum hemorrhage. The pituitary gland, which becomes enlarged during pregnancy, is vulnerable to ischemia and necrosis in the setting of hypotension caused by massive blood loss after delivery. The anterior pituitary is involved most commonly, although posterior gland dysfunction also can occur (1). Improvements in obstetric care have made Sheehan syndrome rare in countries with well-established healthcare infrastructure, although it remains an important cause of hypopituitarism in resource-limited settings (2).

Sheehan syndrome can manifest as partial or panhypopituitarism, with growth hormone, prolactin, and gonadotropins being the most common resulting hormone deficiencies (3, 4). Clinical presentations vary from lactation failure and amenorrhea to adrenal insufficiency and crisis. Acute diagnosis is possible; however, most patients present years after delivery with nonspecific symptoms, such as fatigue or weakness (2, 3). Average delay to diagnosis has been reported as upward of 20 years in multiple retrospective studies (3, 4, 5, 6, 7). Variability and subtlety of presentations are believed to stand in the way of timely diagnosis, giving rise to significant morbidity (1, 2).

We report a 45-year-old woman, gravida 1, para 1, who presented with fatigue 17 years after a postpartum hemorrhage. She was hospitalized for severe hyponatremia and ultimately diagnosed with Sheehan syndrome. The patient gave written informed consent to publish this article.

## Case report

A 45-year-old woman, gravida 1, para 1, with no significant past medical history presented to the emergency department with progressive lightheadedness, dyspnea, nausea, vomiting, and lower extremity myalgias 1 month after immigrating to the United States from China. She also reported a sore throat and cough beginning 3 days prior, followed by 3 episodes of emesis 1 day before presentation. On review of systems, she endorsed chronic fatigue, generalized weakness, pallor, decreased appetite, and brain fog for the past 17 years.

Further history revealed these chronic symptoms began after the birth of her son. At age 28, the patient had a spontaneous term vaginal delivery complicated by severe postpartum hemorrhage. She was told she lost nearly 3 L of blood after delivery due to a retained placenta. She reported losing consciousness, requiring a blood transfusion and hospitalization for 1 week. In the postpartum period, she had agalactia as well as amenorrhea. She recalled taking cyclic progesterone as well as a traditional Chinese medicine to induce bleeding, which was intermittently successful until age 31 when all menstrual-like bleeding stopped despite these medications. She desired additional pregnancies but was unable to conceive again and did not undergo fertility treatment. She was unable to access medical care for comprehensive evaluation of her chronic fatigue, weakness, and brain fog due to financial barriers.

Initial examination was significant for pallor, periorbital edema, and flat affect. She was afebrile with borderline hypotension (90–110/60–70 mmHg) and resting heart rate between 60–70 beats per minute. Height was 1.6 m and weight was 53 kg. Laboratory investigations revealed severe hyponatremia (115 mmol/L) and borderline hypoglycemia (73 mg/dL). Electrocardiogram on admission had prolonged PR and QT intervals.

Further workup revealed undetectable serum cortisol and low adrenocorticotropic hormone (1.9 pg/mL) as well as undetectable thyroxine and inappropriately normal thyroid-stimulating hormone (1.78 uU/mL). Diagnosis was central adrenal insufficiency and central hypothyroidism concerning for panhypopituitarism. The hyponatremia, low blood sugar, and electrocardiographic changes were attributed to these endocrine abnormalities. Myalgias were attributed to hypothyroid myopathy with creatine kinase elevation to 1,680 U/L. Treatment with hydrocortisone 50 mg every 8 hours was initiated on hospital day 1. The next morning, she reported resolution of dyspnea, nausea, vomiting, myalgias, and improvement of fatigue. Treatment with levothyroxine 25 mcg daily was delayed until hospital day 2, after cortisol repletion, as thyroid hormone can worsen cortisol deficiency by increasing its metabolism and clearance.

**Figure 1:**
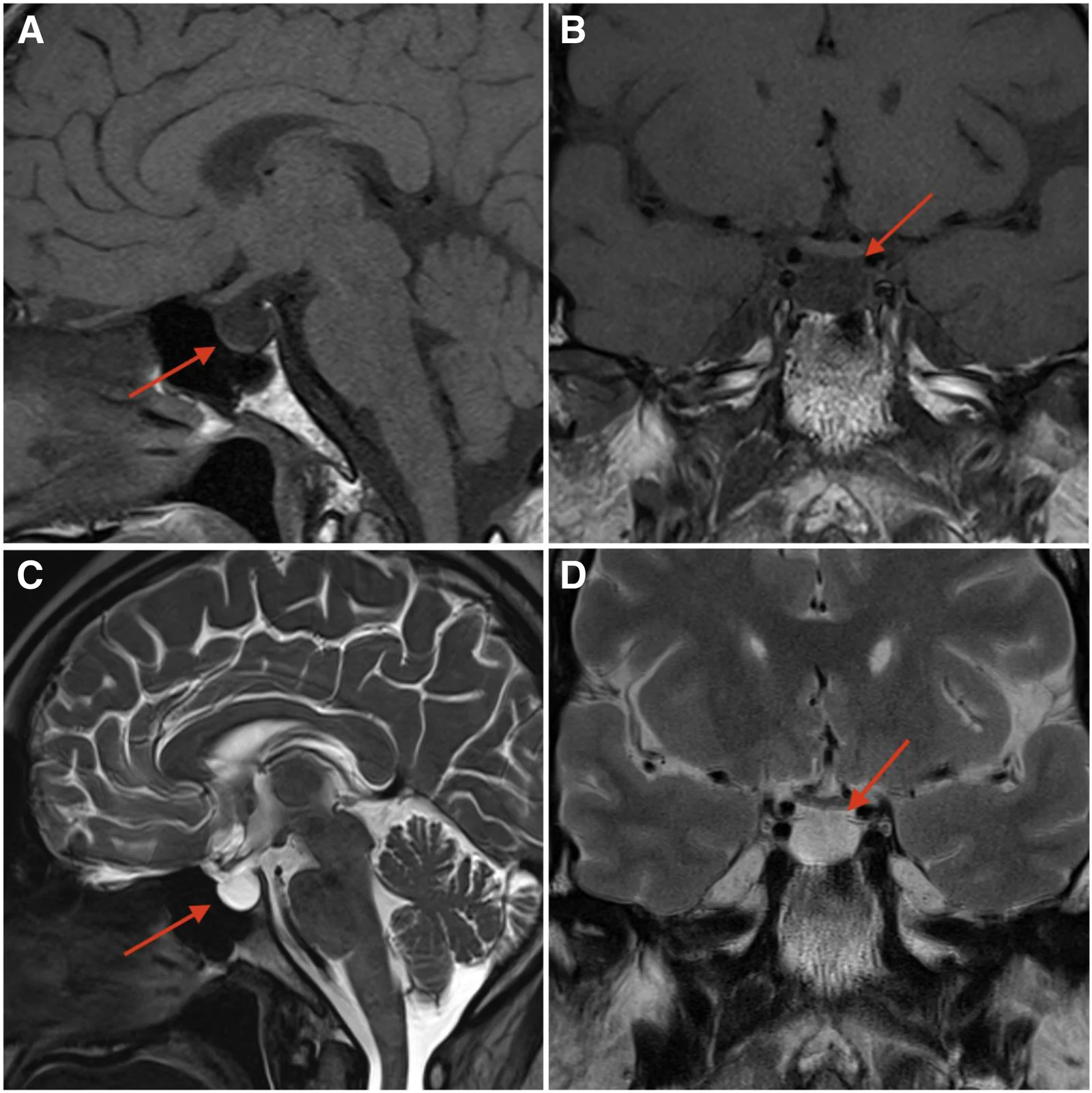
Pituitary magnetic resonance imaging showing an empty sella with mild leftward deviation of the pituitary stalk. T1-weighted sagittal (**A**) and coronal (**B**) sections. T2-weighted sagittal (**C**) and coronal (**D**) sections.

The hyponatremia corrected after treatment of panhypopituitarism with hydrocortisone and levothyroxine. She required multiple boluses of 5% dextrose in water and desmopressin to maintain a safe rate of sodium correction. Contrast-enhanced magnetic resonance imaging revealed a moderately enlarged empty sella with markedly reduced pituitary volume and mild leftward deviation of the pituitary stalk, raising the question of pituitary apoplexy contributing to her pathogenesis (Fig. 1). Additional laboratory results included undetectable immunoglobulim-1, estradiol, and luteinizing hormone levels as well as low prolactin (1.2 ug/L) and follicle-stimulating hormone (2 U/L), further confirming panhypopituitarism consistent with Sheehan syndrome.

After resolution of hyponatremia and marked clinical improvement, the patient was discharged with hydrocortisone 10 mg in the morning and 5 mg in the afternoon, and levothyroxine 50 mcg daily. Close follow-up with endocrinology was arranged for ongoing management.

On endocrinology evaluation 2 weeks after discharge, her energy and weakness had improved, and her thyroxine level had increased to 0.40 ng/dL. She continued to exhibit clinical signs of hypothyroidism, including periorbital edema and pallor. Her levothyroxine dose was increased to 75 mcg daily. Her full, weight-based replacement dose is expected to be 86 mcg (approximately 1.6 mcg/kg/day). She was initiated on cyclic drospirenone-ethinyl estradiol 3-0.02 mg combined oral contraceptive pills (OCPs) and has not had withdrawal bleeding since starting treatment 9 months ago. A dual-energy x-ray absorptiometry scan of the L1-L4 vertebrae (bone mineral density [BMD] 0.821, T-score −2.1, Z-score −1.6), total left hip (BMD 0.726, T-score −1.8, Z-score −1.5), and left femoral neck (BMD 0.589, T-score −2.3, Z-score −1.9) was consistent with osteopenia.

## Discussion

Postpartum hypopituitarism is rare in resource-rich countries with accessible obstetric care as a result of early and proactive interventions to prevent hypovolemic shock in the setting of massive hemorrhage (2). It remains prevalent in places with limited healthcare resources, more commonly seen after home deliveries with reported rates as high as 2.7%–3.9% among parous women of reproductive age (7, 8). However, significant delay to diagnosis is observed in resource-rich and resource-limited settings, indicating pervasive undertreatment and insufficient awareness of the condition (9, 10). In this case, financial barriers to comprehensive follow-up care contributed to diagnosis being delayed nearly 2 decades before immigration to the United States. Thus, a high index of clinical suspicion is indicated particularly in those who have immigrated from under-resourced countries or rural regions, as well as patients who have been out of care.

Hormone deficiencies and subsequent clinical signs and symptoms in Sheehan syndrome evolve gradually after the initial ischemic event (3). The progressive course of hypopituitarism is hypothesized to be secondary to autoimmunity against necrotic pituitary tissue that develops over time, although this has not been established clearly (11, 12). Broadly, acute presentations include agalactia, amenorrhea, and asthenia, whereas delayed manifestations include hyponatremia, hypoglycemia, and adrenal crisis (2, 5). Patients, including ours, present with nonspecific symptoms, such as fatigue, weakness, and anorexia, >50% of the time, which is believed to be the main reason behind diagnostic delay (3) and can result in misdiagnosis as more common syndromes, such as depression or primary hypothyroidism (13). It is possible that the marked worsening of our patient’s clinical course represents progression of pituitary dysfunction as well as an acute exacerbation of chronic adrenal insufficiency and hypothyroidism in the setting of recent travel, stress, and upper respiratory illness.

Sheehan syndrome causes significant morbidity and poor quality of life over time for patients who remain undiagnosed and untreated. Our patient was chronically impaired by debilitating fatigue, brain fog, and weakness for nearly 20 years, through her own early adulthood and her son’s lifetime, a devastating complication of delayed diagnosis that should not go unnoticed.

A better understanding of obstetric factors that predispose to Sheehan syndrome in patients who experience hemorrhage may facilitate more prompt diagnosis. In a small study of massive postpartum hemorrhage, defined as a loss of ≥3 L of blood, 3 of 9 cases had postpartum hypopituitarism. A significant increase in obstetrical disseminated intravascular coagulation score and significant decrease in Glasgow coma score were observed in the Sheehan group. The total volume of hemorrhage and shock index did not differ significantly between groups (14). Further study of predictive factors may enable obstetricians to identify patients at high risk for Sheehan syndrome after a hemorrhage, and also may help uncover the pathogenesis of disease.

Pituitary imaging is important for diagnosing Sheehan syndrome in the chronic period. Partial or complete empty sella appearance on pituitary magnetic resonance imaging has been suggested as an essential criterion for diagnosis in patients with hypopituitarism along with a history of postpartum hemorrhage (1). However, empty sella is a common incidental radiographic finding reported in up to 35% of the general population. In patients without endocrine or neurological abnormalities, empty sella does not have clinical significance. In our patient’s clinical context, her imaging findings are highly suggestive of a secondary etiology of empty sella and support the diagnosis of Sheehan syndrome (15).

The contribution of autoimmunity to progression of Sheehan syndrome has long been discussed but remains uncertain. Multiple retrospective studies have demonstrated the presence of antihypothalamic and antipituitary antibodies in these patients (11, 16). However, a prospective study did not detect antipituitary antibodies in patients with Sheehan syndrome within 6 months postpartum, suggesting any potential role of autoimmunity to be an enhancing rather than etiologic factor of the disease (12). Although our patient’s loss of progesterone withdrawal bleeding over time and later clinical deterioration reflect a progressive course, antibody testing unfortunately was not done in this case.

The mainstay of treatment for postpartum hypopituitarism is hormone replacement with glucocorticoids and levothyroxine, as well as growth hormone in cases of severe deficiency. Gonadal hormones are replaced until the average age of menopause (1). Combined estrogen and progesterone therapy is recommended for patients with an intact uterus. Sequential and continuous regimens are viable strategies in hypopituitarism; current guidelines do not recommend one over the other. In sequential regimens, progesterone withdrawal typically induces bleeding, whereas continuous replacement causes less or no bleeding (17, 18). Although combined OCPs are used commonly in hypopituitarism, they offer nonphysiologic replacement. In comparison, transdermal estrogen can be administered at lower doses than oral preparations, avoiding effects on growth hormone and insulin-like growth factor–1, and thus is recommended for patients with multiple hormone deficiencies (17, 19).

In this case, cyclic combined OCPs were prescribed at the initial endocrinology appointment 2 weeks after her discharge due to insurance constraints. Despite 9 months of this treatment, our patient has not experienced withdrawal bleeding, contributing to a clinical picture of severe hypogonadotropic hypogonadism. A transition to physiologic dosing with transdermal estrogen and intermittent progesterone exposure is planned for a future visit. Supplementation of growth hormone will be considered after optimizing the current replacement regimens.

Timely estrogen and progesterone therapy is recommended in hypopituitarism of all etiologies given evidence for protection of BMD and decreased cardiovascular mortality (20). This is important particularly in the treatment of Sheehan syndrome as patients have demonstrated increased cardiovascular risk as predicted by coronary artery calcium score as well as significantly lower BMD compared with controls (21, 22). In an open-label, nonrandomized study, treatment with a cyclic combined OCP (Ovral-L, ethinyl estradiol 30 μg and levonorgestrel 150 μg) as well as calcium and vitamin D3 for 1 year significantly improved lumbar spine BMD, demonstrating the benefits of hormonal intervention (22).

Although rare, subsequent pregnancy is possible in patients with Sheehan syndrome. There are numerous case reports of successful pregnancies in this group, with many requiring assisted reproductive technology including ovulation induction and in vitro fertilization. With close monitoring and multidisciplinary management, these pregnancies often result in healthy live births (23, 24). This patient was unable to conceive again and did not access fertility treatment. Given the severity of her hormone deficiencies and progression of hypogonadism over time, she would have likely required assisted reproductive technology to conceive.

In conclusion, hypopituitarism is a rare but important complication of postpartum hemorrhage and is widely underdiagnosed. Our case highlights common barriers to timely diagnosis, including nonspecific symptom presentation and limited awareness of the condition. A comprehensive diagnostic approach to evaluation of such symptoms is indicated especially in individuals with a history of postpartum hemorrhage. Without adequate treatment, patients are at risk for substantial morbidity. Prompt diagnosis and initiation of hormone replacement offer an opportunity for marked improvement in quality of life.

## Declaration of Interests

S.G.V. has nothing to disclose. N.J.T. has nothing to disclose. K.S.B. has nothing to disclose. N.A.F. has nothing to disclose. D.C. has nothing to disclose.
